# Editorial: Antibiotic resistance and its continuity in the environmental niche

**DOI:** 10.3389/fvets.2022.1119578

**Published:** 2022-12-20

**Authors:** Marina Spinu, Magdalena Rzewuska

**Affiliations:** ^1^Division of Infectious Diseases, Department of Clinical Sciences, Faculty of Veterinary Medicine, University of Agricultural Sciences and Veterinary Medicine, Cluj-Napoca, Romania; ^2^Institute of Research and Development for Montanology, Cristian-Sibiu, Romania; ^3^Department of Preclinical Sciences, Faculty of Veterinary Medicine, Warsaw University of Life Sciences (WULS), Warszawa, Poland

**Keywords:** antimicrobial resistance, antibiotic stewardship, human, animals, environment niche

Antibiotic resistance is a consequence of biased and exaggerated antibiotic treatments in both humans and animals and has recently been subject of wide-ranging community concern (1). Lack of clinical reasoning and consideration for presence or absence of epidemiological pressure selected representatives of the microbial community showing resistance plasmids looked at as “resistome.” The “resistome” is continuously increasing due to changes in the ultrastructure and subsequently in the metabolism prompted by various factors, due to further introduction of new generation antibiotics and also concurrent influence of other environmental components. Highly performant and rapid laboratory methods are now utmost important for understanding the resistome from a One Health perspective, involving humans, animals and the environment and for its timely diagnosis (2).

Intensive farming technologies for food animals broadly imply the use of antibiotics as therapeutic means, thus trying to reduce the economic and health impact of infectious diseases by diminishing morbidity and mortality. Nevertheless, the spread of antibiotic resistant and MDR bacteria from animal farming to the broader environment may cause diseases in humans, livestock, and wildlife (3).

Research was conducted to clarify the transfer mechanisms of multi drug resistant (MDR) bacteria from farmed animals/food products to humans due to continuously increasing emergence of resistance. The variable potential for innate antibiotic resistance in the soil was also described based on geo-chemical conditions, while bacterio-plankton tolerance to antibiotics in heavy metal polluted areas was highlighted, suggesting the selective importance of pollution in maintaining and spreading antibiotic resistance (4).

Reciprocal relationships that exist between resistant and potentially pathogenic bacteria and their habitat or broader environment need to be precisely defined to allow the development and implementation of preventive and control measures with highest benefits for humans, animals and the environment. A solid connection can be established between the amounts of antimicrobials used and the increase of numbers of bacterial species resistant to them, above the presence of antimicrobial resistance in pristine sources before introduction of antimicrobials in medical and/or farming practice (5).

This Research Topic aimed at updating research results on antibiotic resistance, its emergence and persistence, horizontal transfer of antibiotic resistance genes, rapid diagnosis of multi antibiotic resistance, its prevention and control, and its connections to environmental factors (such as geography, climate, and climate change) as well as the influence of farming and sewage water management, wildlife and its conservation, and others, to have a comprehensive view of the importance of the often disregarded environmental niche in increasing virulence of pathogenic bacteria.

In this special e-collection there are 18 papers covering the above mentioned aspects.

The presence, persistence and technological influences on antimicrobial resistance in domestic animals were the most tackled subjects (10 of 18 papers, 55.55%). Numerous categories of animals from farmed species (pigs, bovine, chickens, and fish) companion animals (dogs, cats, pigeons, and horses), and also wildlife (foxes, seals, and Geoffrey's cats) inhabiting terrestrial and aquatic environments were monitored for the presence of multidrug resistant microbiome. Comprehensive studies of bacteriome resistance in various environments were also published.

Swine industry is a continuously developing economic sector, while there is a constant need and demand for meat and meet products by consumers. Nevertheless, pigs are hosts for numerous zoonotic pathogens, including ported bacteria, whose increasing antimicrobial resistance support their pathogenicity and aggressivenes and thus, their survival in the habitat. Therefore, ubiquitous bacteria such as *Bordetella bronchiseptica* and *Escherichia coli* need close monitoring for their antimicrobial resistance gene profile. The resistance profile found in *B. bronchiseptica* in pigs from China, one of the largest pork producers in the world, was highly variable, but including percentages as high as 83.98 to ampicillin, very commonly used in animal therapy. Over 90% of the isolates were positive to the five virulence factor encoding genes examined, representing a reason for major consumer health concern (Zhang et al.). Similarly, other researchers (Khine et al.) found *mcr* resistance genes to colistin, a last resort antibiotic to fight the infections with *Enterobacteriaceae* in MCRPE (*mcr* positive *E. coli*) isolates also showing MDR and connection to *E. coli* ETEC (enterotoxigenic) pathotype shared by human and animal hosts.

Further, it has been proven that *E. coli* strains originating from non-organic chickens, raised in low-income communities harbor antibiotic resistant genes found in multidrug-resistant and extended-spectrum beta-lactamase (ESBL) phenotypes, which could eventually colonize the human gut of bird contacts or consumers (Murray et al.). In dairy cow herds, mastitis is maybe the most economically impacting disease caused by a variety of agents, with a rapidly changing etiology, frequently involving antibiotic resistant agents, therefore the phenotypic and molecular analysis of *Candida krusei*, a yeast collected from mastitis cases provided valuable information for disease control, indicating that drug-resistance was relying on mutations of the ERG11 gene (Du et al.).

Farming aquatic animals became a widespread practice lately, following the trend of increased protein need for feeding the planet. Due to specific use of antibiotics for microbial disease control, fish farms not only represent a possible source for antimicrobial resistance for consumers but also impact on the environment health, by the location of the ponds either down- or upstream the rivers, thus showing an potentially enhanced multidirectional spread of this resistance. A study carried out in Southern Lithuania, envisaging the simultaneous presence of heavy metal pollution and antimicrobial resistance in the sediment of fish ponds, indicated that in spite of the heavy metal (Co, Cu, and As) levels which did not exceed the maximum allowable concentrations and antibiotic residues (oxytetracycline, florfenicol, and florfenicol amine) present in low amounts or below the detectable limit, the resistance determinants identified (aminoglycoside, β-lactam) create risks to human hosts by potential transfer (Lastauskiene et al.). Experiments aiming at investigating the horizontal transfer of antimicrobial and arsenic resistance genes have provided positive evidence of this between certain bacteria, such as *Rheinheimera* spp. and *E. coli*, suggesting a permanent monitoring of antibiotic/arsenic resistance profile of the bacteriome to reduce or avoid the spread of gene pollutants (Fu et al.).

Similarly, the identification of increase in the CTX-M type ESBL producing *E. coli* variants in the Seine river over time underlined that the aquatic environment exposed to numerous polluting sources, posing risks during recreational activities, is of broad community concern (Girlich et al.).

Several studies mention the MDR or pan-drug resistance in companion animals such as dogs, cats and horses. An extended research of clinical cases in the Iberian peninsula provides an overview of the bacteria most frequently found in dogs and cats and also their resistance profile to antimicrobials, indicating the highest antimicrobial resistance in *Enterococcus* spp. and *Pseudomonas* spp., while interestingly, *Klebsiella* spp., *Proteus* spp. or *Enterobacter* spp. seemed to be the most resistant of *Enterobacteriacea* (Li et al.), when compared to the otherwise MDR ESBL *E. coli*, as indicated by other researchers (Huang et al.). Nevertheless, *Pseudomonas spp*. of canine origin seemed to also be resistant (72.7–100%) to antimicrobials linezolid (LZD) and tigecycline (TGC) efficient in fighting with pan-drug resistant bacteria isolated from humans (Kim and Kim).

A quite widespread category of companion or hobby birds are racing pigeons. Their close contact with humans during feeding, handling and competitions creates the premises for transfer of multidrug resistant bacteria or yeasts (*Staphylococcus aureus*, non-hemolytic *E. coli*, and *C. albicans*) and also the antimicrobial resistance genes to the latter (Chrobak-Chmiel et al.). Further, such birds could, through their interactions and lifestyle, close a loop of antimicrobial resistance in their closer or further environment.

As already mentioned (Li et al.), hospital environment could provide an appropriate environment for the persistence of antimicrobial resistance, not only through the patients seen but also on the contact surfaces. Such a study carried out in an equine hospital consulting local and international patients revealed the presence of multidrug resistant, host-versatile *Salmonella typhimurium* on human and patient contact surfaces, which led back to the significance of biosecurity measures implemented at all times to preserve patient and personnel safety (Soza-Ossandón et al.).

Wildlife, out of direct connection with antibiotic therapy, has been disregarded as a link in the antibiotic resistance transfer chain. Recent research has proven the presence and high incidence (66%) of enterococci in the feces of wild foxes (*Lycalopex gymnocercus*) and Geoffroy's cats (*Leopardus Geoffroyi*) from the Brazilian Pampa, with resistance percentages as high as 94 or 72.6 to rifampicin, one of the most potent broad spectrum antibiotics, and erythromycin, respectively. This phaenomenon was supported by the identified resistance genes (*tet*M/*tet*L and *msr*C/*erm*B) along with virulence genes (*gel*E, *ace*, agg, *esp*, and *cly*A), standing most probably for human interference in the pampa habitat (Oliveira de Araujo et al.).

The marine ecosystem is not spared of antimicrobial resistance, the investigation of marine mammals and costal environment providing valuable information on another direction of antimicrobial resistance spread. As such, the identification of 66.6% MDR of the total *E. coli* isolates from feces of rescued seals, identification of resistance genes in 16 of 39 of the isolates and virulence factors associated with adhesion and siderophores, augmenting the pathogenicity of these strains was relevant (Vale et al.).

A broader study comparing MRSA and MSSA from various animal sources and different environments disclosed clear differences between *mec*C-positive and *mec*C-negative types, with possible human origin of the *mec*C-MRSA including the typically human “immune evasion cluster” (IEC) (Gómez et al.).

A review of the perspectives on antimicrobial resistance at the level middle and low income countries, with special reference to the COVID period, brings forward a pertinent analysis of the antibiotic types toward which the resistance is augmented, the most frequent fields of activity and host species, representing important links within the antimicrobial resistance chain as well as multi-level and multi-actor mitigation strategies (Bandyopadhyay and Samanta).

The food chain represents another potential route for spreading the antimicrobial resistance from farm to fork. Further, the beneficial effects of lactic acid bacteria have been recognized and given attention since decades. Nevertheless, such strains could serve as vehicle for spreading antimicrobial resistance as indicated (Stefańska et al.) during a study which included probiotic feed additives/silage inoculants. Tests carried out on their antibiotic susceptibility/resistance indicated the resistance to aminoglycosides and tetracyclines mainly (26%). Therefore, the authors suggested as a safety criterion the preliminary resistance analysis of the strains of *Lactobacillus* and *Pediococcus* to ensure their appropriate effects. Moreover, the analysis for resistance genes in a non-pathogenic bacteria such as *Bifidobacterium animalis* subsp. *lactis* revealed the presence of *tet*(W) (tetracyclin resistance gene) in 41 of 44 examined strains, but being a part of the ancient resistome, is different from other species and possesses a very low transfer risk (Nøhr-Meldgaard et al.).

In conclusion, the data gathered in the studies and reviews mentioned before provide beneficial information, which, without being exhaustive, offer a valuable insight in the complex matter of antimicrobial resistance and its transmission chain, leaving room for the intriguing and still undiscovered interaction of humans, animals and environment with the aim of preserving One Health.

## Author contributions

Both authors listed have made a substantial, direct, and intellectual contribution to the work and approved it for publication.
